# Defining Phenotypes in Diabetic Nephropathy: a novel approach using a cross-sectional analysis of a single centre cohort

**DOI:** 10.1038/s41598-017-18595-1

**Published:** 2018-01-08

**Authors:** Rosa M. Montero, Athula Herath, Ashfaq Qureshi, Ehsanollah Esfandiari, Charles D. Pusey, Andrew H. Frankel, Frederick W. K. Tam

**Affiliations:** 1 Renal and Vascular Inflammation Section, Department of Medicine, Imperial College London, Hammersmith Hospital, London, W12 0NN UK; 2 Honorary Senior Clinical Lecturer, King’s College, London, UK; 3 Novartis Pharmaceuticals, GU16 7SR Camberley, UK

## Abstract

The global increase in Diabetes Mellitus (DM) has led to an increase in DM-Chronic Kidney Disease (DM-CKD). In this cross-sectional observational study we aimed to define phenotypes for patients with DM-CKD that in future may be used to individualise treatment We report 4 DM-CKD phenotypes in 220 patients recruited from Imperial College NHS Trust clinics from 2004–2012. A robust principal component analysis (PCA) was used to statistically determine clusters with phenotypically different patients. 163 patients with complete data sets were analysed: 77 with CKD and 86 with DM-CKD. Four different clusters were identified. Phenotypes 1 and 2 are entirely composed of patients with DM-CKD and phenotypes 3 and 4 are predominantly CKD (non-DM-CKD). Phenotype 1 depicts a cardiovascular phenotype; phenotype 2: microvascular complications with advanced DM-CKD; phenotype 3: advanced CKD with less anaemia, lower weight and HbA1c; phenotype 4: hypercholesteraemic, younger, less severe CKD. We are the first group to describe different phenotypes in DM-CKD using a PCA approach. Identification of phenotypic groups illustrates the differences and similarities that occur under the umbrella term of DM-CKD providing an opportunity to study phenotypes within these groups thereby facilitating development of precision/personalised targeted medicine.

## Introduction

Diabetes Mellitus (DM) is increasing worldwide and subsequently as people are treated for complications and enjoy longevity, it is inevitable that more people will develop Diabetic Nephropathy (DN). DN has been described since Egyptian times with the last century providing a classification of DN based on albuminuria^1^. The introduction of renin-angiotensin-aldosterone system (RAAS) antagonists in the form of ACEi or ARB, has resulted in the regression of this surrogate marker and slowing of progression of renal dysfunction^2,3^. There is increasing appreciation that DN progression to end-stage kidney disease (ESKD) is not always a stepwise progression through albuminuria with different subgroups progressing at different rates and some progress in the absence of proteinuria, hence the need for us to redefine progression of DN^4^. Progression of the disease and response to the treatment varies in different patients, which may indicate heterogeneity of diabetes chronic kidney disease (DM-CKD). DM-CKD may consist of different sub-population and phenotypes which may require different treatment approaches. In doing so we should be able to identify personalised targeted therapies for people with this potentially devastating disease.

Appreciation of heterogeneous disease subgroups has previously been described in Asthma, with distinct subgroups^5^ with a set of reference clinical endpoints. These subgroups have been shown to have physiologically distinct underlying processes that have facilitated the rational use of targeted therapy^6,7^. Targeted therapy can be used to specifically target pathways of the disease thereby avoiding the common clinical endpoint. This has led to a revolution in treatment for certain subgroups of this disease^8^. Clustering methods have been applied to the respiratory epidemiological field and perceived as ‘steps in the right direction’^9,10^ with the discovery of these subgroups.

Porrini, *et al*.^11^, recently described non-proteinuric pathways in patients with type 2 DM (T2DM) associated with loss in renal function thereby illustrating phenotypic spectrum of DM that is independent of proteinuria. Given that patients with and without proteinuria with DM may develop ESKD, a new method looking at the spectrum of people with DM-CKD is needed^12^. The aims of this study were to 1) identify new phenotypes in DM-CKD and 2) compare this with CKD caused by other renal diseases using clinical variables and cytokines to ascertain whether there are more specific markers than albuminuria to determine who will progress to ESKD.

## Objectives

Determine whether clinical variables may identify heterogeneous subgroups within a cohort of patients with DM-CKD to facilitate further study of underlying mechanisms leading to progression to ESKD which may lead to novel treatment approaches for different sub-groups of DM-CKD.

Define and characterise subgroups within the diabetic nephropathy cohort to act as a template for further studies.

### Research Design and Methods

All methods were performed in accordance with current study guidance and regulations. Following ethics and research and development study approval (NRES Committee London-West London & GTAC 04/Q0406/25) participants with diabetic nephropathy or renal disease without diabetes mellitus were recruited prospectively from renal clinics at Imperial College Healthcare NHS Trust Hospitals London UK, between 2004 to 2012. In this study CKD is used to describe patients with renal disease without DM. Because of the risk/benefit balance, only a limited proportion of patients with DM-CKD had a kidney biopsy (5 patients) with CKD controls having 62 biopsy proven diagnosis. The remainder of the CKD group was diagnosed with imaging or ultrasound showing small kidneys not amenable to renal biopsy. The diagnosis of DN was made by an elevated uACR on at least two occasions or reduction in eGFR and the exclusion of other aetiologies for CKD by history, clinical, and laboratory examinations, including autoantibody screening, urine sediment and renal imaging. Patients with CKD without DM were classified as the control CKD group. The diagnoses of the non-diabetic CKD group are shown in Supplementary Table 2. Some of these renal diseases had patients on immunosuppressive treatments and the details of this were recorded to determine whether immunosuppression had an effect within the CKD groupings. CKD controls were used to determine whether there were any differences specific to DM-CKD in the cytokines tested in this study.

Informed consent was obtained prior to any participants entering the study. Participants consenting to enter the prospective cohort study had their clinical and biochemistry data captured using questionnaires at baseline. Biochemistry data included MDRD GFR, serum albumin, urea, creatinine, haemoglobin, HbA1C, total cholesterol and C-reactive protein (CRP). Plasma and urinary samples were collected, processed and stored for cytokine analysis at baseline. Cytokines previously described in DN and studied by our group were analysed; monocyte chemoattractant protein-1 (MCP-1)^13^, C-Chemokine ligand-18 (CCL18)^14^ and macrophage migration inhibitory factor (MIF)^15^. All of these cytokines have been reported to be raised in DN and may play an important role in pathogenesis of the disease, hence they were chosen to determine whether these change in the different phenotypes and whether they are more specific to DM-CKD than CKD. These cytokines are immunomodulatory and may provide further insight as to whether there are specific changes in different phenotype. Exclusion criteria included: those receiving dialysis therapy or with kidney, pancreas or kidney pancreas transplants, those under 18 years old; and those unable to consent. See supplementary for details of ELISA protocols.

### Data Availability

All data analysed during this study are included in this published article and the Supplementary Information files.

Participant selection was unbiased and reflects the widespread presentations of diabetic nephropathy to the renal clinic. However selection bias exists as these patients were already diagnosed with CKD or DM-CKD and hence referred to the specialist renal clinics.

### Quantitative variables

MCP-1, CCL18 and MIF levels were measured in plasma and urinary samples by ELISA. Details of antibodies used in Table S2 of the Supplementary.

### Statistical methods

We report cross-sectional analysis of baseline data in CKD and DM-CKD. We collected 43 variables to begin with and these were reduced to 30 in order to optimize the utility and exclude variables with predominantly missing data in the total dataset for principal component analysis (See Table S3 Supplementary for Variables used). Principal component analysis (PCA) was performed on these variables as a method for reducing interaction between variables. Then, cluster analysis based on the main components of the PCA was performed to search for DM-CKD phenotypes (Tables 1 and 2).

**Table 1:** The characteristics of the individual variables studies within the phenotypes 1 to 4 - Continuous variables.

**Phenotype (MEAN/SD)**	**1**	**2**	**3**	**4**	**p**
Number of patients per Phenotype	34	40	43	46	
Urine CCL18 Creatinine ratio ng/mmol	1.15 (3.20)	4.89 (11.36)	10.86 (33.01)	4.00 (6.89)	0.145
Serum CCL18 ng/ml	140.16 (108.66)	138.50 (79.05)	154.06 (92.64)	137.43 (77.36)	0.86
Urine MCP 1 Creatinine ratio ng/mmol	12.38 (13.78)	26.76 (36.27)	23.32 (26.64)	25.54 (41.45)	0.231
Serum MCP 1 ng/ml	0.26 (0.11)	0.35 (0.63)	0.25 (0.10)	0.25 (0.16)	0.474
Urine MIF Creatinine ratio ng/mmol	266.29 (574.23)	353.94 (701.88)	596.99 (656.69)	476.48 (725.60)	0.165
Serum MIF ng/ml	1921.37 (1960.24)	5629.28 (9664.91)	4593.94 (3307.73)	3853.52 (4098.75)	0.077
Age	60.97 (11.04)	64.55 (12.20)	61.86 (15.64)	50.87 (13.17)	<0.001
Weight Kg	88.80 (24.13)	89.18 (17.40)	80.28 (19.49)	78.54 (18.34)	0.023
Height cm	168.54 (9.22)	169.22 (9.98)	167.11 (7.24)	167.76 (9.64)	0.851
Body mass index (BMI)	31.10 (7.45)	31.32 (5.06)	30.24 (6.01)	28.10 (5.68)	0.154
Duration of Diabetes	17.59 (11.86)	21.76 (8.35)	2.16 (5.87)	0.28 (1.28)	<0.001
HbA1C	8.06 (1.20)	8.31 (1.67)	6.36 (1.14)	6.57 (2.46)	<0.001
Haemoglobin (Hb) g dL	13.25 (1.43)	11.97 (1.80)	12.62 (1.79)	13.15 (1.63)	0.007
C-reactive protein (CRP) mg L	4.91 (5.52)	4.65 (8.77)	3.16 (5.78)	2.76 (5.04)	0.344
Total Cholesterol mmol/L	4.95 (1.56)	4.25 (1.09)	4.56 (0.76)	5.49 (1.36)	<0.001
Albumin g/L	38.00 (3.29)	36.23 (4.23)	38.56 (3.20)	38.93 (6.32)	0.037
Urea mmol/L	6.84 (2.12)	16.26 (6.51)	17.42 (7.32)	5.83 (1.98)	<0.001
Serum Creatinine umol/L	112.59 (34.21)	222.10 (84.62)	226.58 (102.99)	100.74 (21.13)	<0.001
Baseline GFR MDRD ml min 1 73sq m	64.39 (22.17)	29.25 (10.49)	27.29 (9.73)	67.75 (15.89)	<0.001
Systolic BP	152.94 (24.67)	133.97 (18.05)	133.95 (16.91)	132.89 (17.76)	<0.001
Diastolic BP	82.94 (11.52)	69.92 (9.83)	76.21 (12.55)	81.35 (14.30)	<0.001
Urinary Albumin Creatinine ratio mg/mmol	47.56 (70.83)	67.94 (74.48)	45.76 (64.17)	33.60 (76.11)	0.176
Urinary Protein Creatinine ratio mg/mmol	88.65 (111.53)	104.60 (112.08)	59.66 (69.71)	53.26 (96.01)	0.324

**Table 2:** The characteristics of the individual variables studies within the phenotypes 1 to 4 - Categorical variables.

**Phenotype**	**1**	**2**	**3**	**4**	**p**
Number of patients per Phenotype	34	40	43	46	
Diabetes Type (%)					<0.001
No Diabetes	0 (0.0)	0 (0.0)	34 (79.1)	43 (93.5)	
Type 1	2 (5.9)	4 (10.0)	0 (0.0)	0 (0.0)	
Type 2	32 (94.1)	36 (90.0)	9 (20.9)	3 (6.5)	
Gender = Female (%)	9 (26.5)	9 (22.5)	20 (46.5)	26 (56.5)	0.003
Ethnicity (%)					0.004
White	5 (14.7)	15 (37.5)	28 (65.1)	25 (54.3)	
Black	8 (23.5)	10 (25.0)	7 (16.3)	10 (21.7)	
Asian	18 (52.9)	13 (32.5)	7 (16.3)	8 (17.4)	
Chinese	1 (2.9)	1 (2.5)	0 (0.0)	0 (0.0)	
Other	2 (5.9)	1 (2.5)	1 (2.3)	3 (6.5)	
Smoking Habits (%)					0.173
Non-smoker	20 (58.8)	19 (48.7)	12 (54.5)	28 (73.7)	
Ex-smoker	4 (11.8)	2 (5.1)	2 (9.1)	4 (10.5)	
Current Smoker	10 (29.4)	18 (46.2)	8 (36.4)	6 (15.8)	
Retinopathy (%)					<0.001
None	20 (58.8)	10 (25.0)	42 (97.7)	46 (100.0)	
Background	1 (2.9)	2 (5.0)	0 (0.0)	0 (0.0)	
Pre-proliferative	2 (5.9)	3 (7.5)	0 (0.0)	0 (0.0)	
Proliferative	10 (29.4)	24 (60.0)	1 (2.3)	0 (0.0)	
End-stage diabetic eye disease	1 (2.9)	1 (2.5)	0 (0.0)	0 (0.0)	
Neuropathy (%)					<0.001
None	34 (100.0)	27 (67.5)	43 (100.0)	46 (100.0)	
Autonomic	0 (0.0)	6 (15.0)	0 (0.0)	0 (0.0)	
Peripheral	0 (0.0)	7 (17.5)	0 (0.0)	0 (0.0)	
Cerebrovascular accident (CVA) (%)	4 (11.8)	3 (7.5)	0 (0.0)	0 (0.0)	0.022
Ischaemic heart disease (IHD) (%)	13 (38.2)	13 (32.5)	8 (18.6)	6 (13.0)	0.03
Peripheral vascular disease (PVD) (%)	1 (2.9)	14 (35.0)	0 (0.0)	1 (2.2)	<0.001
History of Renovascular Disease (%)					0.565
0	34 (100.0)	38 (95.0)	41 (95.3)	45 (97.8)	
Single kidney	0 (0.0)	1 (2.5)	2 (4.7)	1 (2.2)	
Both kidneys	0 (0.0)	1 (2.5)	0 (0.0)	0 (0.0)	
Urine infection (%)	3 (8.8)	3 (7.5)	4 (9.3)	2 (4.3)	0.811
**Medications**					
Insulin (%)	21 (61.8)	34 (85.0)	1 (2.3)	0 (0.0)	<0.001
Metformin (%)	13 (38.2)	1 (2.5)	0 (0.0)	2 (4.3)	<0.001
Sulphonylurea (%)	7 (20.6)	7 (17.5)	6 (14.0)	0 (0.0)	0.021
PPAR.agonist (%)	6 (17.6)	2 (5.0)	1 (2.3)	1 (2.2)	0.017
ACE inhibitor (ACEi) (%)	18 (52.9)	26 (65.0)	15 (34.9)	16 (34.8)	0.012
Angiotensin 2 Receptor blocker (ARB) (%)	18 (52.9)	16 (40.0)	22 (51.2)	14 (30.4)	0.13
Statin (%)	24 (70.6)	35 (87.5)	24 (55.8)	20 (43.5)	<0.001
Vitamin D supplementation (%)	0 (0.0)	9 (22.5)	10 (23.3)	3 (6.5)	0.004
On immunosuppression (%)	0 (0.0)	0 (0.0)	6 (14.0)	15 (32.6)	<0.001

**Figure 1:**
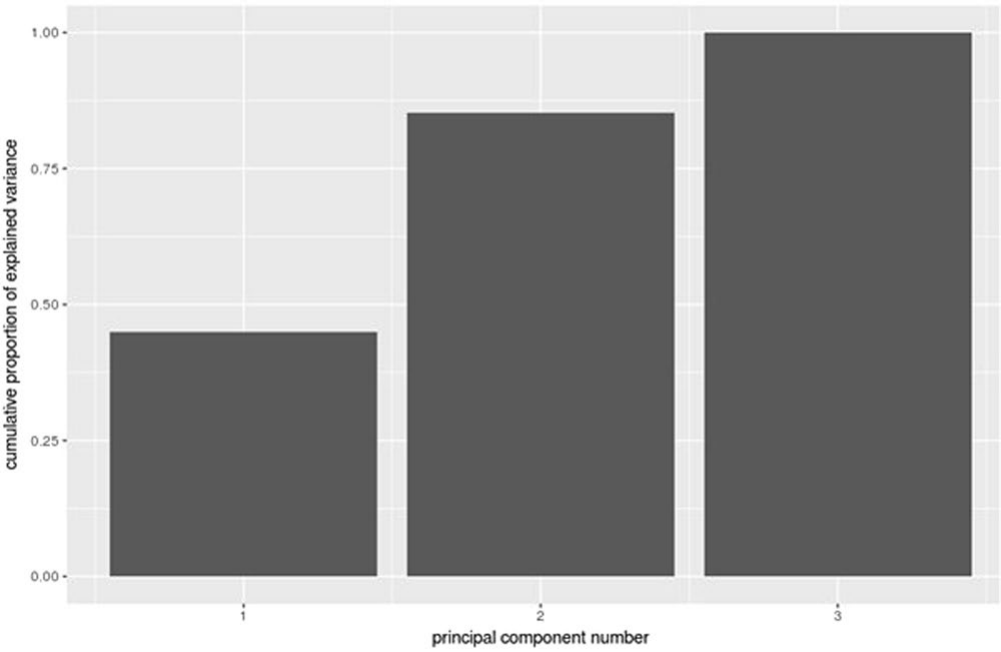
Cumulative plot of 3 Principal components Principal components (PC) PC1 is aligned to traditional complications of diabetes with ethnicity being one of the major variables in this domain. PC2 major variables include; Baseline GFR, Urea, Urinary Albumin/Creatinine Ratios. PC3 major variables of diastolic and systolic blood pressure suggesting a cardiovascular domain.

**Figure 2:**
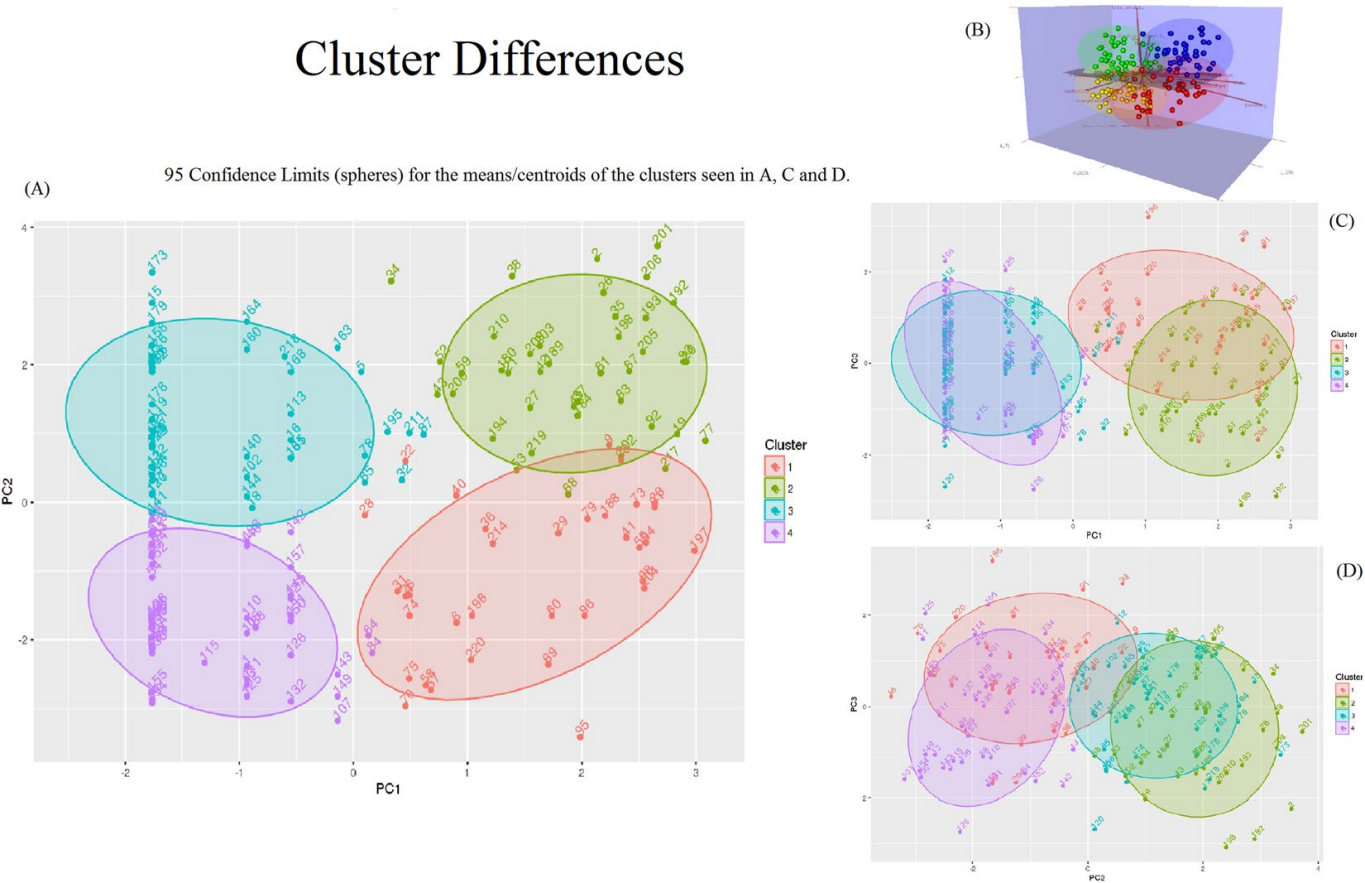
Phenotypes arising from Principal components The four phenotypes are illustrated by the different clusters illustrated. Cluster 1 -Cardiovascular phenotype, Cluster 2 -Advanced CKD with traditional microvascular complications phenotype, Cluster 3 -Advanced CKD with inflammatory cytokine profiling phenotype and Cluster 4 -Younger hypercholesterolaemic phenotype. The clusters Fig (**A**), (**C**) and (**D**) are illustrating their movement according to the PC. (**B**) Illustrates the clusters 3-dimensionally.

**Figure 3:**
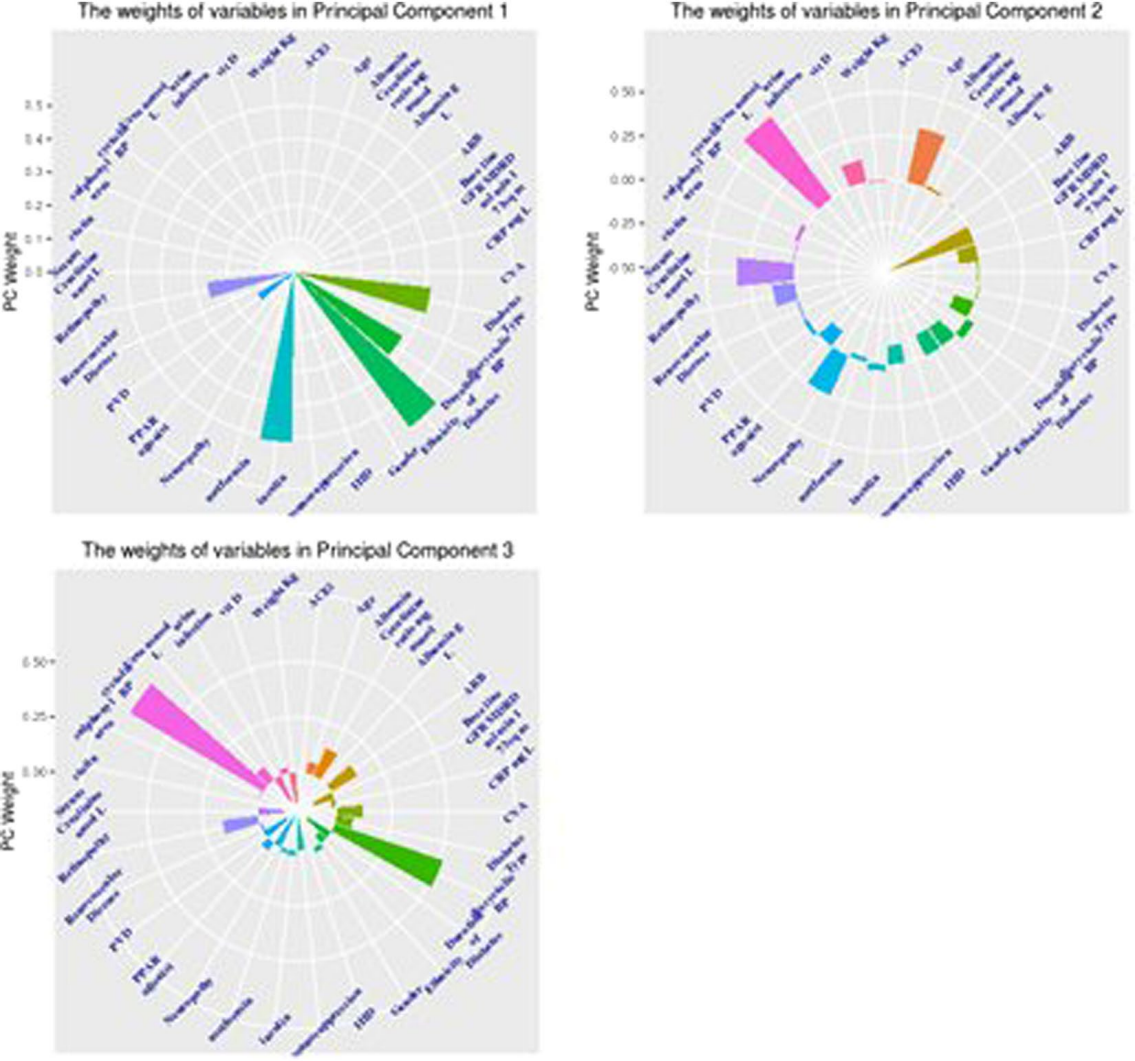
The weights of the variables in Principal Component 1, 2 and 3. The outer circle depicts the positive weights while the inner circle depicts the negative weights.

We normalised variables with BOX-COX transformation and scaled and centred the variables using the caret package^16^ of statistical system R^17^. We then applied robust principal components analysis as described in^18^ using package pcAPP^19^ of R statistical system. This captured the total variation of the dataset with three principal components (Fig. 1- Cumulative plot) by reducing the dimensions from 30 to 3. We then applied k-means clustering using package cluster^20^ of R statistical system on the principal components (PCs) and derived four clusters or Phenotypes. The spatial distribution of patients across the PCs, are described in PCA plots (PC1 vs PC2, PC1 vs PC3 and PC2 vs PC3) (see Fig. 2). The characteristics of the PCs in terms of the weights of individual variables are shown in the plots (Fig. 3). Then cluster analysis based on the main components of the PCA was performed to search for DN phenotypes.

### Results

220 patients were recruited for the study with 163 patients with full clinical and biomarker datasets. There were 99 males and 64 females. The ethnic mix was reflective of the local population with 73 white, 35 black, 46 South Asian, 2 Chinese and 7 Other. 77 patients had CKD alone and 86 had DM-CKD. The DM-CKD group had 6 T1DM patients and 80 T2DM patients.

## Disease domains of CKD and DM-CKD

The process of deriving principal components (PCs), reducing the original 30 variables into 3 high level variables (PC1, PC2 and PC3), simultaneously creates equivalents of major traits or dimensions of the underlying dataset, in our case CKD and DM-CKD. The PC1, PC2 and PC3 establish a new coordinate system to describe the CKD and DM-CKD patients; the PCs can be considered as disease ‘forces’ that act on individual CKD and DM-CKD patients. In Agusti *et al*.^21^ terminology these form the treatable traits of the DM-CKD. Each trait/PC can be explained by the weights of the original variables within the PC. The weights of each PC, are shown in the Fig. 3. The outer circle depicts the positive weights while the inner circle depicts the negative weights.

Closer examination of these diagrams reveals three traits in CKD and DM-CKD. The first trait/PC is aligned to traditional complications seen with long term diabetes such as insulin use, retinopathy, duration of diabetes, and peripheral vascular disease, with ethnicity also being one of the major variables in this domain. The second trait/PC has major variables determining the severity of CKD including: baseline GFR, Urea, Urine Albumin/Creatinine ratios. The third trait/PC with major variables of diastolic and systolic blood pressure may suggest a disease domain of a cardiovascular renal CKD type.

## Disease Phenotypes of CKD and DM-CKD

As described in the methods section, the principal components were subjected to K-Means cluster analysis. The cluster analysis resulted in four clusters (Fig. 2) with the predominant variables seen in Table 1. Each such cluster may form a phenotype. Differences between the clusters are illustrated in the Supplementary graphs. The overlaps between the clusters are visualised in Fig. 2. Cluster 1 and 2 comprise DM-CKD patients, with clusters 3 and 4 being predominantly CKD without DM, with few DM-CKD. DM-CKD patients are seen in all four clusters, with each cluster providing a different phenotype (Table 2).

### Phenotype 1 – Cardiovascular phenotype

Phenotype 1 consists of 34 patients all of whom have DM-CKD. This phenotype has a significantly higher systolic and diastolic blood pressure than the other phenotypes. The total cholesterol is high in this group compared with those in phenotype 2 despite a high use of statins. HbA1c levels were comparable to phenotype 2 and therefore cannot be the differentiating factor for the different phenotypes: 8.31% (67.3 mmol/mol) phenotype 1 and 8.06% (64.6 mmol/mol) phenotype 2. Albuminuria or proteinuria did not significantly affect the phenotyping of DM-CKD (Table 1b). The mean GFR in this phenotype was 64.4mls/min/1.73 m^2^ compared with phenotype 2, whose GFR was 29.3mls/min/1.73 m^2^. Phenotypes 3 and 4 were predominantly CKD patients that showed a similar grouping of renal function as that seen in phenotypes 1 and 2. GFR for phenotype 3 and 4 was 27.3mls/min/1.73 m^2^ and 67.8mls/min/1.73 m^2^, respectively. The different phenotypes of patients with DM-CKD are evident as there were also DM-CKD patients in phenotypes 3 and 4. Urinary and serum MCP-1, CCL18 and MIF data did not reach significant differences between the phenotypes yet each group had a different cytokine profile that requires larger numbers to confirm these preliminary findings (see supplementary for cytokine profile graphs for the different DM-CKD phenotypes).

### Phenotype 2 – Advanced CKD with traditional microvascular complications phenotype

Phenotype 2 was entirely composed of DM-CKD patients, 40 in total with significantly worse GFR than phenotype 1. This group had a longer duration of diabetes compared with phenotype 1, mean 21.76 years compared to 17.59 years, respectively; however this was not significant and was not a determinant of the clustering. Those in phenotype 2 were more anaemic compared with those in phenotype 3, who had similar renal function (Table 1a).

Phenotype 1 and 2 had more ischaemic heart disease (IHD) and stroke (CVA) than phenotypes 3 and 4 that were predominantly CKD. Angiotensin converting enzyme inhibitors and statins were used more in phenotypes 1 and 2 compared with 3 and 4.

### Phenotype 3 – Advanced CKD with inflammatory cytokine profiling phenotype

Phenotype 3 is predominantly CKD alone with 9 DM-CKD that all had T2DM with a single individual on insulin and the remaining 8 on oral hypoglycaemics. This phenotype had a mean GFR of 27.3mls/min/1.73 m^2^, similar to phenotype 2 but without microvascular complications. The DM-CKD patients have a shorter duration of diabetes in phenotype 3 and anaemia was less prominent compared to phenotype 1 and 2. Patients in phenotypes 3 and 4 with CKD alone are also on immunosuppression unlike phenotypes 1 and 2. Urinary MIF and serum and urinary CCL18 were higher in this phenotype however this was not significantly different and a larger study is required to determine whether this profile is specific to this phenotype (Table 1a).

### Phenotype 4 – Younger hypercholesteraemic phenotype

Phenotype 4 has 3 patients with DM-CKD all of whom have T2DM with the shortest duration of DM in this cohort. GFRs in this cohort have a mean of 67.75mls/min/1.73 m^2^ that is similar to phenotype 1. The mean age is significantly younger at 50.9 years and the cardiovascular phenotype is limited to hypercholesterolemia. Urinary MIF levels are higher than phenotype 1 and 2 (Table 1a).

### Conclusions

This study illustrates a novel approach to characterise DM-CKD patients in two ways. First, we introduce novel thinking on the variability of the disease using high level disease traits (PC1, PC2 and PC3) and these alone may form a basis for treatable traits in DM-CKD^21^. Second, we derive four phenotypes of DM-CKD patients (Phenotype 1 to 4) in a well characterised population. To our knowledge this is the first time this approach has been used to establish the treatable traits and phenotypes in adults with DM-CKD. We are reporting preliminary results and suggest further exploration of both the treatable traits and the phenotypes to derive effective and high precision treatment modalities in DM-CKD.

This was a predominantly male cohort with phenotypes 3 and 4 having more females than phenotypes 1 and 2. Phenotype 1 appears to have predominantly South Asian patients with phenotype 2 including a wide ethnic variety. Phenotypes 3 and 4 had predominantly more white people however this cohort had larger numbers of white people and thus with a larger cohort an ethnic and gender effect may be determined.

Microvascular complications were more commonly seen in phenotype 2, where a longer duration of diabetes was reported. A prospective study is needed to determine whether individuals stay in these phenotypes or move from one phenotype to another with the progression of their disease. This will be established in the following study that will allow identification and recruitment of patients into these phenotypes with genetic and molecular mapping further characterising patients in these phenotypes. The follow-up will elucidate whether these phenotypes are fixed or whether people move between phenotypes allowing further mapping of the disease. The phenotypes, however, show that despite the similar GFRs in the DM-CKD and CKD population there are differences that arise in people with diabetes that are specific and individual to the person.

The variability in the phenotype of patients with DM-CKD is illustrated by patients with DM-CKD found in all four phenotypes, albeit a predominance in phenotypes 1 and 2. The spectrum of phenotypes within this disease has been captured using the principal component analysis and as a concept this may explain the overlap we see between the traditional T1DM and T2DM classification, whilst also providing a possible explanation for people responding well to intensive glycaemic and blood pressure control whilst others with DM-CKD continue to progress to ESKD despite this. Access to increasing genome, molecular, cellular and tissue data will further allow specific pathways to be identified in different subgroups of patients with DM-CKD, similar to those seen in asthma. Further large cohorts are required to determine whether these phenotypes, based on predominantly clinical variables, are conceptually robust with a further overlay of genomic, molecular and proteomic data. This may lead to further elucidation of predominant signalling pathways that may subsequently be targeted more effectively in the right population.

This study’s phenotypes were derived from 3 PCs/traits from the PCA that included the long term complications of diabetes (Trait 1), the severity of CKD (Trait 2) and the cardiovascular burden in CKD (Trait 3). Further clarification of these traits may reveal treatable traits (18) in CKD and DM-CKD that may lead the way in changing how we treat these patients.

The limitations to this study include: the small number of T1DM patients (6 patients) recruited to the cohort and lack of long term follow up to determine whether these phenotypes are predictive of outcome or whether the phenotype changes with duration of the disease. The small proportion of T1DM in comparison to T2DM is a reflection of the common pattern in general nephrology clinics. Therefore, the main driver for identification of DM-CKD phenotypes were data from T2DM patients (80/86). T1DM patients have been included to suggest that a similar model and statistical approach can be used for a larger T1DM data set in future studies. At present no conclusion can be made to compare T1DM and T2DM patients because of the small size of T1DM patients A further study recruiting more T1DM will determine how similar or different the DM-CKD entity is between T1DM and T2DM and whether a common pathway really exists between the two or whether there is a T1DM DM-CKD and T2DM DM-CKD that do not share phenotypes. More patients are also needed in each phenotype to determine whether the high urinary MIF levels seen in phenotype 4 remains in view of the small sample size of DM-CKD in this group. A further limitation to this study is the lack of histopathology renal biopsies in patients within the different DM-CKD phenotypic groups which is limited by current clinical practice and concern on the risk/benefit ratio in patients with DM. DM-CKD has traditionally been a kidney disease that has been clinically diagnosed in view of the risk of renal biopsy however there is an increasing appreciation amongst the renal community of the important role of renal biopsy in further understanding the disease process and facilitating a more personalised medical treatment approach.

In addition, many CKD patients are on immunosuppression that may have influenced the clusters and the cytokine biomarkers assessed in this study. The follow-up of this study is in progress and we hope to report our findings soon. A longer study is planned to increase number of patients with these characteristics to confirm these phenotypes.

We believe that we are the first group to describe different phenotypes in DN using a PCA approach. Our results may form a platform to use a combination of clinical variables and cytokines to group patients with predominant phenotypes. This approach allows a structure to combine more clinical data and biological results to determine different endotypes within the phenotypes identified. A similar approach has been used in Asthma and has led to the discovery of specific phenotypes that have subsequently been amenable to more effective targeted personalised therapy. We hope this approach will help map further understanding of DN in a structured way whereby biomarkers may reflect disease progression within these groups.

## Electronic supplementary material

Supplementary Material
