# Supplementary material for: Defining Phenotypes in Diabetic Nephropathy: a novel approach using a cross-sectional analysis of a single centre cohort
Source: Sci Rep. 2018 Jan 8;8:53. doi: 10.1038/s41598-017-18595-1 (PMC5758706; doi:10.1038/s41598-017-18595-1)
Supplement: Supplementary file 1 — Supplementary Material [file 41598_2017_18595_MOESM1_ESM.pdf]

Defining Phenotypes in Diabetic Nephropathy: a novel approach using a cross-sectional analysis of a single centre cohort.

Short running title: **Defining phenotypes in Diabetic Nephropathy**

Rosa M Montero (MBBS, MD)[1,2], Athula Herath (PhD)[3], Ashfaq Qureshi (MBBS, MMED SCi, Mphil)[1], Ehsanollah Esfandiari (MD, PhD)[1], Charles D Pusey (DSc)[1], Andrew H Frankel (MD)[1], Frederick W K Tam (MBBChir, PhD)[1]

Affiliations
[1]Renal and Vascular Inflammation Section, Department of Medicine, Imperial College London, Hammersmith Hospital, London W12 0NN, UK
[2]Honorary Senior Clinical Lecturer, King's College London
[3]Novartis Pharmaceuticals, GU16 7SR, UK

Corresponding author: Dr RM Montero, Nephrology Consultant, UK, Honorary Senior Clinical Lecturer King's College London, rosa_montero@hotmail.com

Supplementary Materials

MCP-1 ELISA protocol

A 96 well plate is covered with 100μl of MCP-1 Capture antibody (MAB679) per well.

Plate covered overnight in the fridge

The plate is washed three times with washing solution (PBS/Tween). A total volume of 3 times 360μl/w

300μl of blocking solution is added to each well and the plate is covered for 1 hour.

Standards are prepared. MCP-1 Standard (279-MC).

Preparation of standards 5μl of Stock to 5000μl of Diluent concentration

10000pg/ml (label as Standard)

11 tubes are labelled with the following concentrations (pg/ml);

5000, 2500, 1250, 625, 312.5, 156.25, 78.125, 39.06,

19.53, 9.76, 0

500μl of Diluent is added to each tube

500μl from each tube labelled Standard is added to tube

labelled 5000 pg/ml and then to tube 2500 pg/ml continuing serial

dilution except for tube 0 where none is added.

Tubes labelled 2500 pg/ml to 0 are used

Prepare samples

Wash plate 3 times 360µl/well

Add 100µl Standards to the standard part of the plate

Add 100µl of samples to the remaining empty wells of the plate

Cover the plate for 2 hours rotating on a slow shaker

Take out the MCP-1 Detection antibody (BAF279) and prepare 22µl in 11mls of Diluent

Wash the plate 3 times 360µl/well

Add 100µl of Detection Antibody to each well.

Cover the plate for 2 hours

Take out the substrate from the fridge

Wash plate 3 times 360µl/well

Prepare Streptavidin 2µl in 20mls PBS

Add Streptavidin to the plate 100µl/well and cover for 20 minutes

Prepare Substrate 5 minutes before use (before washing with Streptavidin). Add 5.5ml of A to 5.5ml B

Wash the Streptavidine 3 times 360µl/well

Add 100µl Substrate to each well of the plate

Cover and protect from direct sunlight for 5-30 minutes until the 2500pg/ml Standard becomes deep blue.

Prepare Stop Solution (10mls H_2SO4 into 90mls of water)

Set up the ELISA plate reader for 450nm wavelength

Add 50µl Stop Solution to each well of the plate and mix gently – samples and standards will turn yellow in colour

Read the plate in the ELISA plate reader

<u>CCL18/PARC ELISA protocol</u>

A 96 well plate is covered with 100µl of CCL18 Capture antibody (MAB394) per well. 22µl Capture antibody in 11ml in PBS.

Plate covered overnight in the fridge

The plate is washed three times with washing solution (PBS/Tween). A total volume of 3 times 360µl/w

300µl of blocking solution is added to each well. The plate is covered for 1 hour.

Standards are prepared. CCL18 Standard 5µl in 12.5ml of Diluent (394-PA).

Preparation of standards 5µl of Stock to 5000µl of Diluent concentration

10000pg/ml (label as Standard)

11 tubes are labelled with the following concentrations (pg/ml);

5000, 2500, 1250, 625, 312.5, 156.25, 78.125, 39.06,

19.53, 9.5, 0

500µl of Diluent is added to each tube

500µl from each tube labelled Standard is added to tube

labelled 5000 pg/ml and then to tube 2500 pg/ml continuing serial

dilution except for tube 0 where none is added.

Tubes labelled 5000 pg/ml to 0 are used

Prepare samples

Wash plate 3 times 360µl/well

Add 100µl Standards to the standard part of the plate

Add 100µl of samples to the remaining empty wells of the plate

Cover the plate for 2 hours rotating on a slow shaker

Take out the CCL18 Detection antibody (BAF394) and prepare 22µl in 11mls of Diluent

Wash the plate 3 times 360µl/well

Add 100µl of Detection Antibody to each well.

Cover the plate for 2 hours

Take out the substrate from the fridge

Wash plate 3 times 360µl/well

Prepare Streptavidin 2µl in 20mls PBS

Add Streptavidin to the plate 100µl/well and cover for 20 minutes

Prepare Substrate 5 minutes before use (before washing with Streptavidin). Add 5.5ml of A to 5.5ml B

Wash the Streptavidin 3 times 360µl/well

Add 100µl Substrate to each well of the plate

Cover and protect from direct sunlight for 5-30 minutes until the 5000pg/ml Standard becomes deep blue.

Prepare Stop Solution (10mls H_2SO4 into 90mls of water)

Set up the ELISA plate reader for 450nm wavelength

Add 50µl Stop Solution to each well of the plate and mix gently – samples and standards will turn yellow in colour

Read the plate in the ELISA plate reader

<u>Human MIF duoset (DY289) ELISA protocol</u>

A 96 well plate is covered with 100µl of MIF Capture antibody (Part 840489) per well.

Plate covered overnight in the fridge

The plate is washed three times with washing solution (PBS/Tween). A total volume of 3 times 360µl/w

300µl of Reagent Diluent is used as the blocking solution and added to each well. The plate is covered for 1 hour.

Standards are prepared. MIF Standard (Part 840491).

Preparation of standards 40µl of Stock to 560µl of Diluent concentration

2000pg/ml (label as Standard)

8 tubes are labelled with the following concentrations (pg/ml):

2000, 1000, 500, 250, 125, 62.5, 31.25, 0

300µl of Reagent Diluent is added to each tube

300µl from each tube labelled Standard is added to tube

labelled 2000 pg/ml and then to tube 1000 pg/ml continuing serial

dilution except for tube 0 where none is added.

Tubes labelled 2000 to 0 are used

Prepare samples

Wash plate 3 times 360µl/well

Add 100µl Standards to the standard part of the plate

Add 100µl of samples to the remaining empty wells of the plate

Cover the plate for 2 hours rotating on a slow shaker

Take out the MIF Detection antibody (Part 840490) and prepare 38.9µl in 7mls of Diluent

Wash the plate 3 times 360µl/well

Add 100µl of Detection Antibody to each well.

Cover the plate for 2 hours

Take out the substrate from the fridge

Wash plate 3 times 360µl/well

Prepare Streptavidin HRP 100µl in 20mls Reagent Diluent

Add Streptavidin HRP to the plate 100µl/well and cover for 20 minutes

Prepare Substrate 5 minutes before use (before washing with Streptavidin HRP). Add 5.5ml of A to 5.5ml B

Wash the Streptavidin HRP 3 times 360µl/well

Add 100µl Substrate to each well of the plate

Cover and protect from direct sunlight for 5-30 minutes until the 2500pg/ml Standard becomes deep blue.

Prepare Stop Solution (10mls H_2SO4 into 90mls of water)

Set up the ELISA plate reader for 450nm wavelength

Add 50µl Stop Solution to each well of the plate and mix gently – samples and standards will turn yellow in colour

Read the plate in the ELISA plate reader

Table 1 All chemokine Abs were purchased from R&D systems (Abingdon, UK).

**ELISA**	**Details**	**[Capture Ab]**	**[Detection Ab]**	**Sensitivity**	**[Highest Standard]**

hMIF	Murine monoclonal anti-human MIF Ab for capture and biotinylated goat anti-human MIF Ab for detection. E.coli derived recombinant human MIF from R&D systems was used as the antigen for the standard curve. The standard curve ranged from 31.25-30000pg/ml.	2µg/ml	0.1µg/ml	31.25pg/ml	30000pg/ml
MCP-1	Murine monoclonal anti-human MCP-1 Ab for capture and biotinylated goat anti-human MCP-1 Ab for detection. E.coli derived recombinant human MCP-1 from R&D systems was used as the antigen for the standard curve. The standard curve ranged from 9.8-2500pg/ml.	2µg/ml	0.1µg/ml	9.8pg/ml	2500pg/ml
CCL18	Murine monoclonal anti-human Ab for capture and biotinylated goat anti-human CCL18 Ab for detection. E.coli derived recombinant human anti CCL18 from R&D systems was used as the antigen for the standard curve. The standard curve ranged from 19.5-5000pg/ml.	2µg/ml	0.1µg/ml	19.5pg/ml	5000pg/ml

Table 2 – Diagnosis of renal disease in CKD

**Non DM-CKD**	**Immunosuppressive treatment**	**Number of patients on immunosuppression**
Renal Vasculitis	Yes	16
Lupus Nephropathy	Yes	12
Minimal change disease	Yes	2
Focal segmental glomerulosclerosis	No	3
Membranous glomerulonephritis	Yes	3
IgA nephropathy	Yes	5
Polycystic kidney disease	No	0
Nephrectomy post renal tumours	No	0
Reflux nephropathy	No	0
Granulomatous interstitial nephritis	Yes	1
Renal artery stenosis	No	0
Hypertensive nephropathy	No	0
Sarcoidosis	Yes	1
Lithium toxicity	No	0
Chronic scarring on biopsy	No	0

Table 3 – Variables collected from renal cohort

Variables
Diabetes (Type1 or Type 2)
Gender
Age
Ethnicity
Duration of Diabetes
Systolic blood pressure
Diastolic blood pressure
Weight (Kg)
Urea (mmol/L)
Serum Creatinine (umol/L)
Baseline MDRD GFR (ml/min/1.73sqm)
Albumin (g/L)
C-reactive protein (CRP) (mg/L)
Urinary albumin Creatinine Ratio (mg/mmol)
Urine infection
Retinopathy
Neuropathy
Cerebrovascular accident (CVA)
Ischaemic heart disease (IHD)
Peripheral vascular disease (PVD)
Renovascular Disease
Insulin
Metformin
Sulphonylurea
PPAR.agonist
Angiotensin 2 receptor blocker (ARB)
Vitamin D supplementation
On immunosuppression
Angiotensin converting enzyme inhibitor (ACEi)
Statin

Supplementary Figure 1: Graphs depicting the different Serum and Urinary Cytokines profiles: MCP-1, CCL18 and MIF, respectively in the four different DM-CKD phenotypes found on the x axis. The results were presented as mean and (standard error)

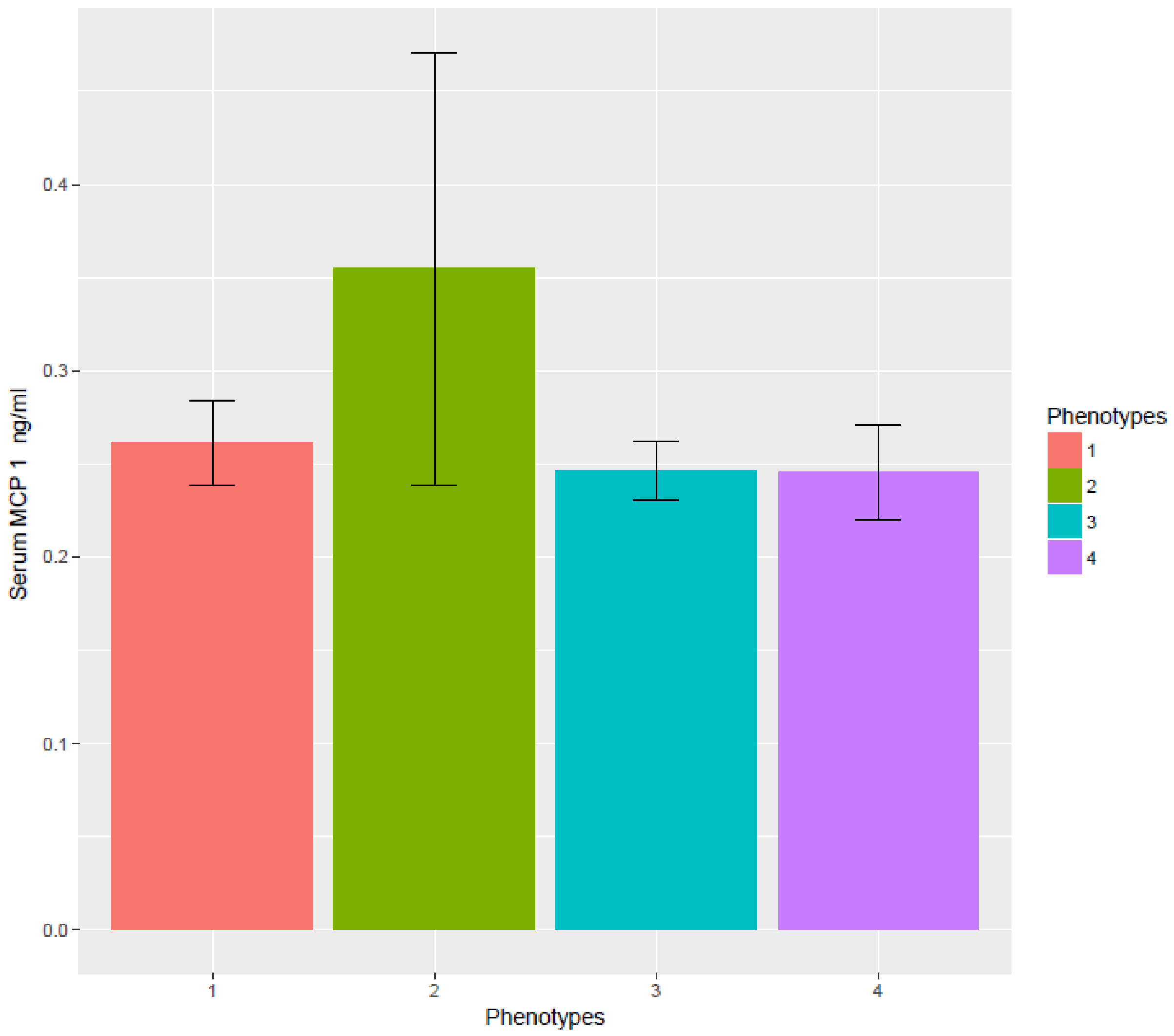

Serum MCP 1 ng/ml
0.0
0.1
0.2
0.3
0.4
1
2
3
4
Phenotypes
Phenotypes
1
2
3
4

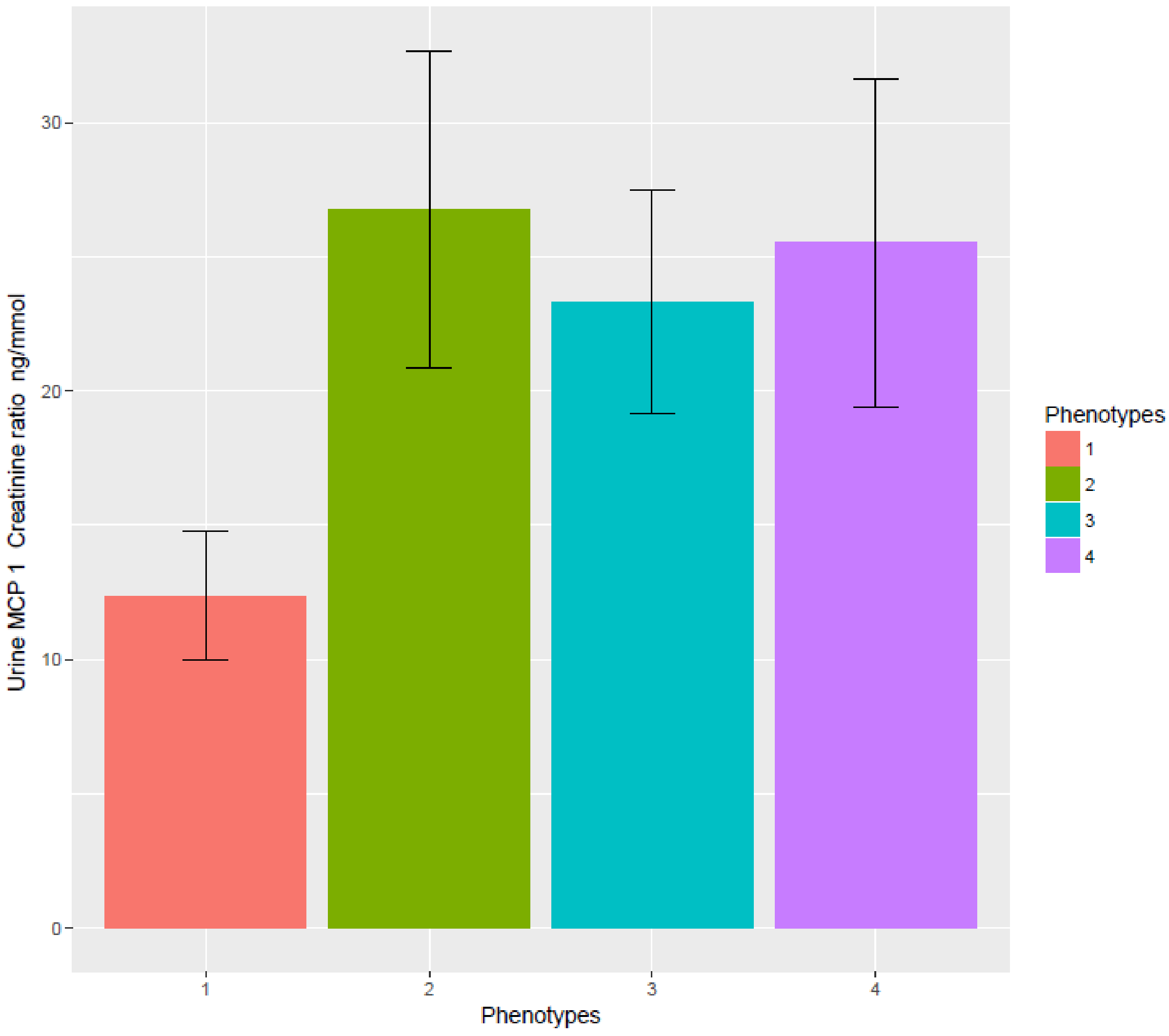

Urine MCP 1 Creatinine ratio ng/mmol
30
20
10
0
1
2
3
4
Phenotypes
Phenotypes
1
2
3
4

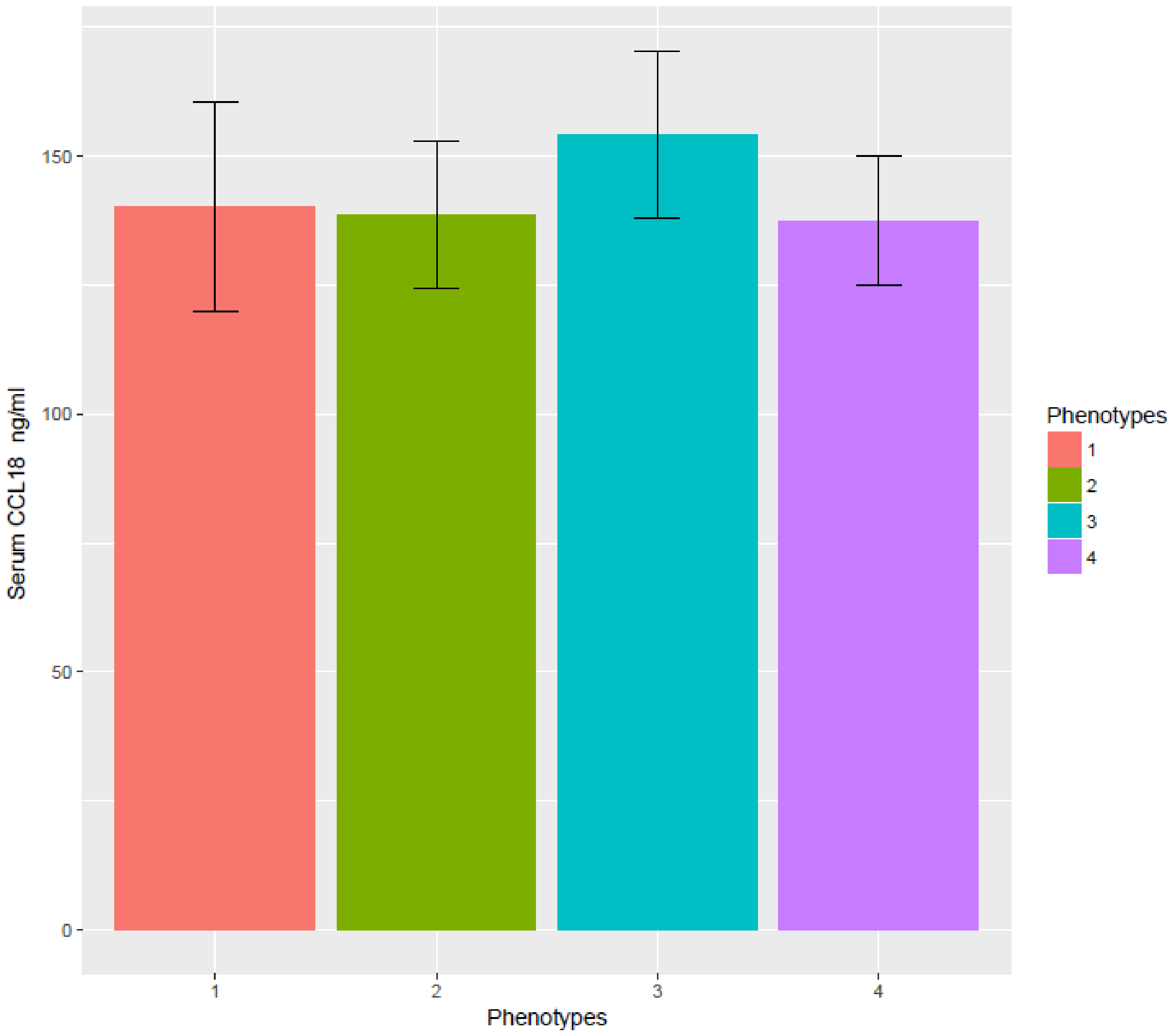

Serum CCL18 ng/ml
150
100
50
0
1
2
3
4
Phenotypes
Phenotypes
1
2
3
4

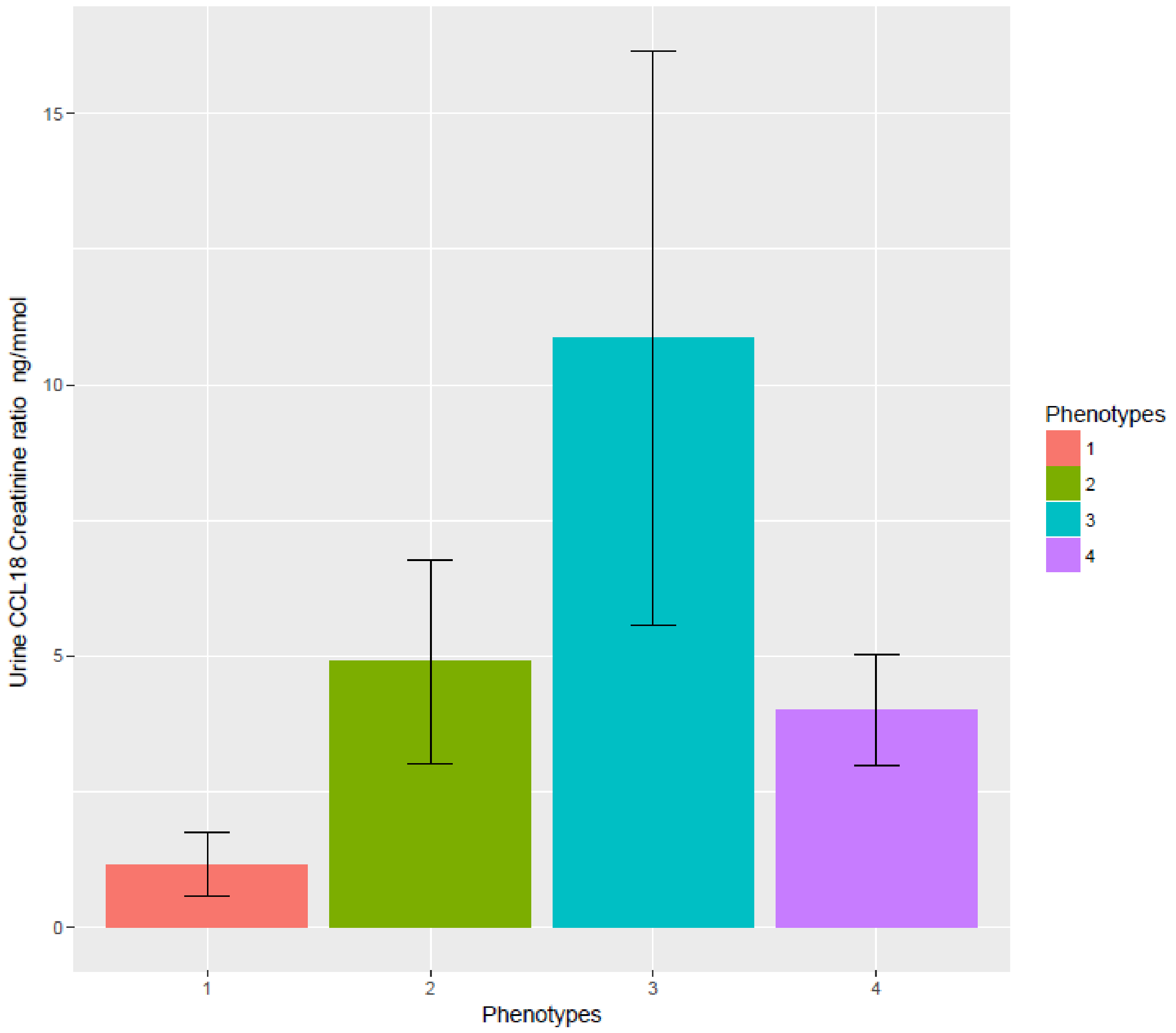

Urine CCL18 Creatinine ratio ng/mmol
15
10
5
0
1
2
3
4
Phenotypes
Phenotypes
1
2
3
4

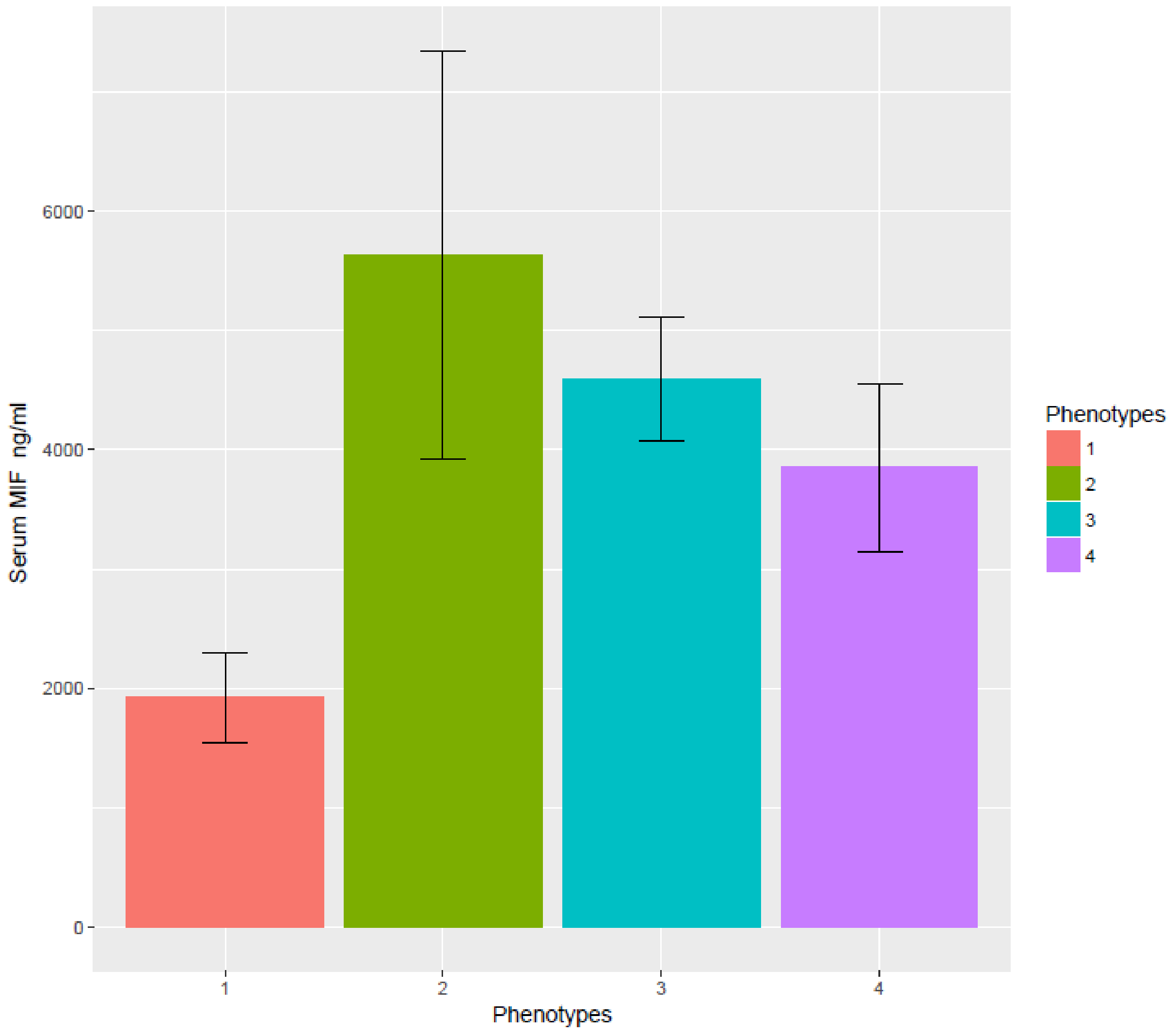

Serum MIF ng/ml
6000
4000
2000
0
1
2
3
4
Phenotypes
Phenotypes
1
2
3
4

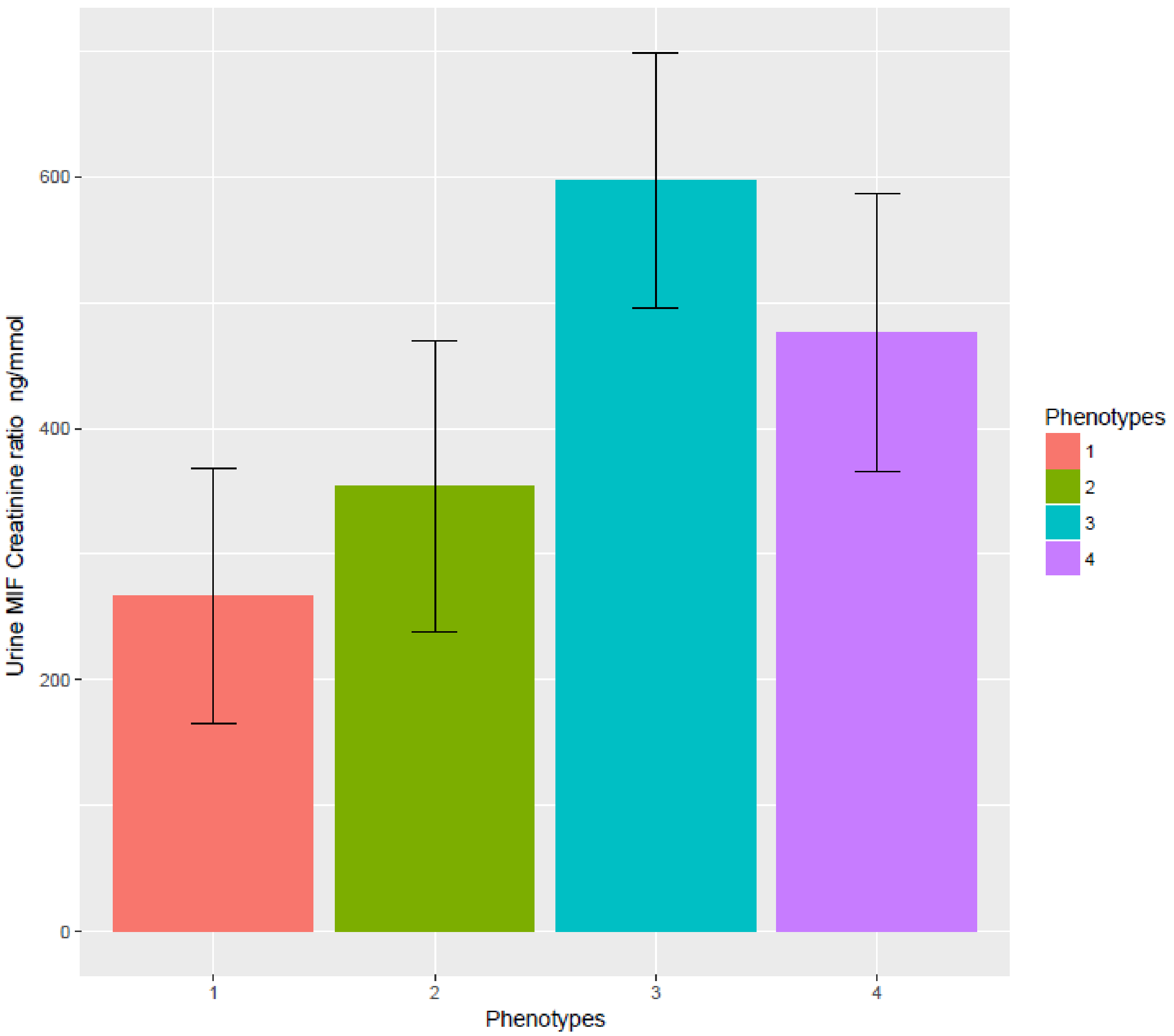

Urine MIF Creatinine ratio ng/mmol
600
400
200
0
1
2
3
4
Phenotypes
Phenotypes
1
2
3
4
